# Myelin Peptide–Mannan Conjugate Multiple Sclerosis Vaccines: Conjugation Efficacy and Stability of Vaccine Ingredient

**DOI:** 10.3390/vaccines9121456

**Published:** 2021-12-08

**Authors:** John Matsoukas, George Deraos, Kostas Kelaidonis, Md Kamal Hossain, Jack Feehan, Andreas G. Tzakos, Elizabeth Matsoukas, Emmanuel Topoglidis, Vasso Apostolopoulos

**Affiliations:** 1 Drug Discovery Laboratory, NewfvDrug, P.C., Patras Science Park, 26504 Patras, Greece; deraos@gmail.com (G.D.); k.kelaidonis@gmail.com (K.K.); bethmatsouka@gmail.com (E.M.); 2 Institute for Health and Sport, Victoria University, Melbourne, VIC 3030, Australia; md.hossain18@live.vu.edu.au (M.K.H.); jack.feehan@vu.edu.au (J.F.); 3 Department of Physiology and Pharmacology, Cumming School of Medicine, University of Calgary, Calgary, AB T2N 4N1, Canada; 4 Department of Chemistry, University of Ioannina, 45110 Ioannina, Greece; atzakos@uoi.gr; 5 Materials Science Department, University of Patras, 26504 Patras, Greece; etop@upatras.gr; 6 Immunology Program, Australian Institute for Musculoskeletal Science (AIMSS), Melbourne, VIC 3021, Australia

**Keywords:** mannan, MOG_35–55_, MBP_83–99_, PLP_131–149_, myelin, epitope, peptide, (KG)_5_, conjugation, quantification, stability, UPLC-MS/MS, HPLC, electrochemistry

## Abstract

Myelin peptide–mannan conjugates have been shown to be potential vaccines in the immunotherapy of multiple sclerosis. The conjugates are comprised from the epitope peptide and the polysaccharide mannan which transfers as a carrier the antigenic peptide to dendritic cells that process and present antigenic peptides at their surface in complex with MHC class I or class II resulting in T-cell stimulation. The conjugation of antigenic peptide with mannan occurs through the linker (Lys–Gly)5, which connects the peptide with the oxidized mannose units of mannan. This study describes novel methods for the quantification of the vaccine ingredient peptide within the conjugate, a prerequisite for approval of clinical trials in the pursuit of multiple sclerosis therapeutics. Myelin peptides, such as MOG_35–55_, MBP_83–99_, and PLP_131–145_ in linear or cyclic form, as altered peptide ligands or conjugated to appropriate carriers, possess immunomodulatory properties in experimental models and are potential candidates for clinical trials.

## 1. Introduction

Multiple sclerosis (MS) is an inflammatory autoimmune-mediated disorder of the central nervous system involving a complex immune activation response, including T cells, B cells, and pro-inflammatory cytokines which attack the myelin sheath [1,2,3,4,5,6,7]. As such, there is demyelination of neurons resulting in axonal loss, neuronal functionality, and disability [8]. Extensive studies on the mechanism of multiple sclerosis pathogenesis targeting treatment have been reported [9,10,11,12,13,14]. The course of MS follows four different clinical patterns: (i) relapsing-remitting MS (accounting for 80–90% of MS cases), (ii) progressive relapsing MS (accounting for 10–20% of MS cases), (iii) secondary progressive MS (not so common), and (iv) primary progressive MS [15,16,17]. The primary cause of the disease is unknown, but a complex inflammatory cascade is associated at the site of demyelination, with predominantly autoimmune CD4+ T cells, interferon gamma-producing T helper (Th1) cells, Th17 cells, and B cells. Autoimmune T cells have been identified and shown to recognize peptide fragments from the main proteins of the myelin sheath including myelin basic protein (MBP), proteolipid protein (PLP), and myelin oligodendrocyte glycoprotein (MOG). In humans, the immunodominant epitopes that are recognized by CD4+ Th1 cells are MBP_83–99_, PLP_139–151_, and MOG_35–55_. These epitopes have been used as tools in our and other studies that have led to the development of a series of mutated linear and cyclic peptide analogues, studied in animal models, and evaluated for immunomodulation in peripheral blood mononuclear cells from patients with MS [18,19,20]. These studies have shown that cyclic analogues are more stable to enzymatic proteolysis, bind well to major histocompatibility class II molecules, and specifically antagonize Th1 cells, representing good candidates for their evaluation as immune modulators against MS [18]. A peptide on its own is not fully immunogenic and requires a carrier to deliver it to antigen-presenting cells.

We have shown that mannan, a poly-mannose carbohydrate isolated from the cell wall of yeast when conjugated to cancer proteins, induces Th1 or Th2 immune responses depending on the mode of conjugation and has therapeutic benefits against cancer when conjugated to peptides, proteins, and DNA. In humans, mannan conjugated to MUC1 protein induces immune responses, clinical responses in adenocarcinoma patients, and protection against cancer recurrence [21]. In addition, mannan has the added advantage, as it stimulates dendritic cells via Toll-like receptor 4. In MS, mannan conjugated to linear or cyclic MOG, MBP, or PLP immunodominant peptides or as altered peptide ligands thereof, results in diversion of Th1 to Th2 immune responses [18,19,20]. The conjugation process, however, of mannan to a peptide is much more complex than its conjugation to protein, as the conjugation reaction occurs via lysine residues. As proteins are large complex structures, often having numerous lysine residues, conjugation completion is often achieved; however, short peptides often do not contain multiple lysines, and conjugation efficacy is limited. As such, a small lysine linker comprising five lysine–glycine amino acids (Lys–Gly)_5_ or (KG)_5_ [5,19] has been developed, enabling effective conjugation between mannan and peptides. In these studies, a method for estimating the complete conjugation of peptide to mannan and its stability over time is crucial for translating this platform into human clinical trials.

The analysis and identification of myelin epitopes or their mutants in polysaccharide–peptide conjugates require specialized techniques that differ significantly from those methods used for small molecules. In this study, a novel method was developed that confirms the total conjugation of polysaccharide mannan with the antigenic peptide MOG_35–55_. The MOG_35–55_ peptide was used as an example peptide in this study. This study allowed us to accurately evaluate the stability of the peptide component in the conjugate using HPLC and electrophoresis techniques. Electrochemical methods, such as cyclic voltammetry (CV), were also applied

These combined techniques were used both for the qualification and quantification of conjugated peptides. These included identifying unknown compounds, determining the isotopic composition of elements in a molecule, and determining the structure of a compound by noting its fragmentation. Other uses included quantifying the amount of a peptide in a peptide-conjugate sample.

In our studies, these techniques were used efficiently to quantify and estimate the stability of the peptide component in the peptide–mannan conjugates, which could be used for assessing potential conjugations for human clinical trials. Herein, we used UPLC-MS/MS techniques to analyze the fragments produced by hydrolysis with specific Glu-C enzymes of peptide MOG_35–55_ synthesized with (KG)_5_ at its N-terminus ((KG)_5_-MOG_35–55_) and of the conjugate mannan–(KG)_5_-MOG_35–55._ The peptide and the conjugate were hydrolyzed with endo glutamate peptidase enzyme, which cleaves the peptides at the Glu-C site. In particular, we focused on identifying a specific fragment of MOG_37–55_ expected from hydrolysis with this enzyme. In both cases, cleavage of the Glu-C peptide bond afforded a fragment of MOG_37–55_ identified by mass spectrometry. The fragment’s peptide peak was identical with the synthetic peptide MOG_37–55_. (MS^+^ 367, 584) confirming fragmentation and cleavage at the Glu-C site.

It is noteworthy that the MBP_82–98_ epitope peptide has been clinically tested in phases I, II, and III and was found safe even if the trial was suspended due to the lack of efficacy. The study was performed for the first time using a peptide epitope on patients with secondary progressive MS (MAESTRO-01 study) [22,23]. MBP–mannan conjugates have been investigated and were found to be potential vaccines in the treatment of MS [18,19,20].

## 2. Materials and Methods

### 2.1. Preparation of the Mannan–Peptide Conjugate

Preparation of mannan in its oxidized form was performed using our previously published method. The MOG_35–55_ peptide (ChinaPeptides, Shanghai, China) was conjugated into oxidized mannan via the (KG)_5_ linker, which was synthesized together with the MOG_35–55_ peptide at the N-terminus. Briefly, 1 mg of (KG)_5_-MOG_35–55_ peptide was added to a solution of oxidized mannan (in bicarbonate buffer pH 9.0) and allowed to react for up to 30 h at 23 °C in the dark, taking sample aliquots at different time points to monitor conjugation completion and characterization.

### 2.2. Monitoring of the Conjugation by HPLC

We used a Waters 2695 HPLC (Alliance) system using a photodiode array detector. A Lichrosorb RP-18 reversed-phase analytical column (C18 35 μm, 4.6 × 50 mm PIN 186003034). Analysis was achieved with a stepped linear gradient of solvent A (0.08% TFA in 100% H_2_O) and in solvent B (0.08% TFA in 100% acetonitrile) over 30 min with a flow rate of 3 mL/min. The reaction of oxidized mannan with a (KG)_5_-MOG_35–55_ peptide was monitored by HPLC. The (KG)_5_-MOG_35–55_ HPLC peak disappeared within six hours, indicating completion of the conjugation. The samples were kept for 3 years and assessed by HPLC for stability.

### 2.3. Preparation of the Electrodes and Monitoring of the Conjugation Cyclic Voltammetry

The graphite/SiO_2_ film electrodes were prepared using our previously published method [24]. Electrochemical experiments were performed on an Autolab PGSTAT-101 potentiostat (Metrohm, Herisau, Switzerland). The electrochemical cell was a 10 m, three-electrode stirring cell made of glass with a Teflon cap employing a platinum wire as the counter electrode, an Ag/AgCl in 3.5 M KCl reference electrode, and a graphite/SiO_2_ film on ITO conducting glass as the working electrode. The electrolyte, an aqueous solution of 10 mM NaH_2_PO_4_ (pH 7), was thoroughly deaerated by bubbling with argon prior to the experiments, and an argon atmosphere was kept during the electrochemical measurements. The measured potentials were recorded with respect to the reference electrode, and all experiments were carried out at room temperature.

## 3. Results

### 3.1. Conjugation of the (KG)_5_-MOG_35–55_ Peptide with Mannan

**Figure 1:**
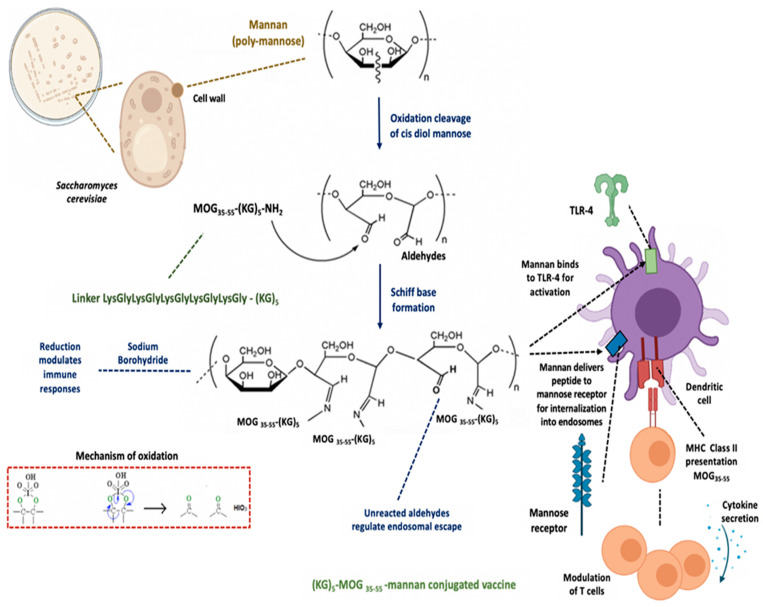
The mannan carrier conjugated to the peptide via (KG)_5_ linker, resulting in the regulation of immune cells. In particular, the mannan conjugation of an epitope peptide with oxidized mannan offers T-cell tolerance and immune regulation by preventing or controlling disease through the release of appropriate cytokines. Figure drawn in part by Biorender.com (https://biorender.com/, accessed on 6 December 2021). MOG, myelin oligodendrocyte; KG, lysine–glycine; MHC, major histocompatibility complex; TLR, Toll-like receptor.

Quantification of the peptide in the peptide–mannan conjugates was required by the authorities for the candidate to reach clinical phase 1 evaluation. Figure 1 shows, schematically, the conjugation of the (KG)_5_-MOG_35–55_ with the oxidized mannan.

**Figure 2:**
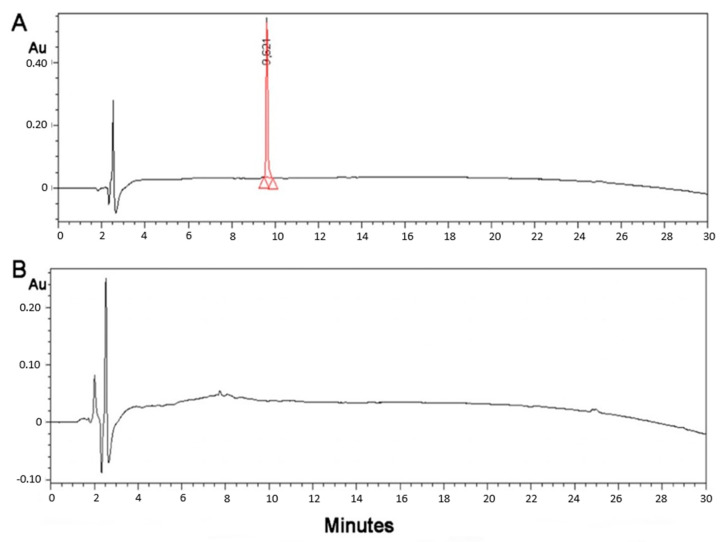
HPLC graph of the conjugation reaction shows the starting material peptide (KG)_5_-MOG_35–55_ at (**A**) zero time of the reaction and (**B**) after 6 h showing complete conjugation. There was no free peptide and no fragmentation of the peptide (adapted from our previous paper [24]).

**Figure 3:**
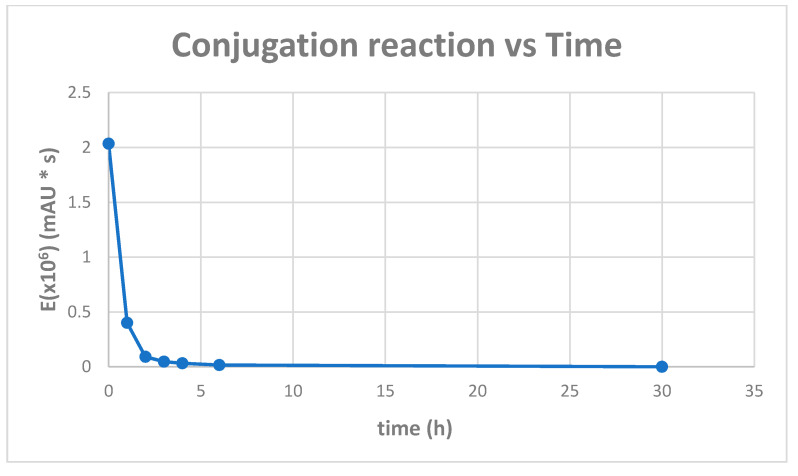
Conjugation reaction versus time, and the time course of the conjugation reaction between the oxidized mannan and the (KG)_5_-MOG_35–55_. The MOG_35–55_ HPLC peak disappeared within 6 h showing complete conjugation within this timeframe.

The conjugation of the (KG)_5_-MOG_35–55_ peptide with the oxidized mannan occurred through lysine side-chains of the (KG)_5_ linker via Schiff’s base reaction as shown by the gradual loss of the (KG)_5_-MOG_35–55_ peptide peak at a retention time (RT) of 9.621 in the HPLC analysis (Figure 2). Figure 2 shows the HPLC chromatograph of the conjugation completion. The reaction between (KG)_5_-MOG_33_-_55_ and oxidized mannan was quick, and the reaction was completed within 6 h. Figure 3 shows the completion of the conjugation reaction versus time.

### 3.2. Attempt to Conjugate MOG_35–55_ Peptide with Oxidized Mannan without (KG)_5_ Was Not Successful

**Figure 4:**
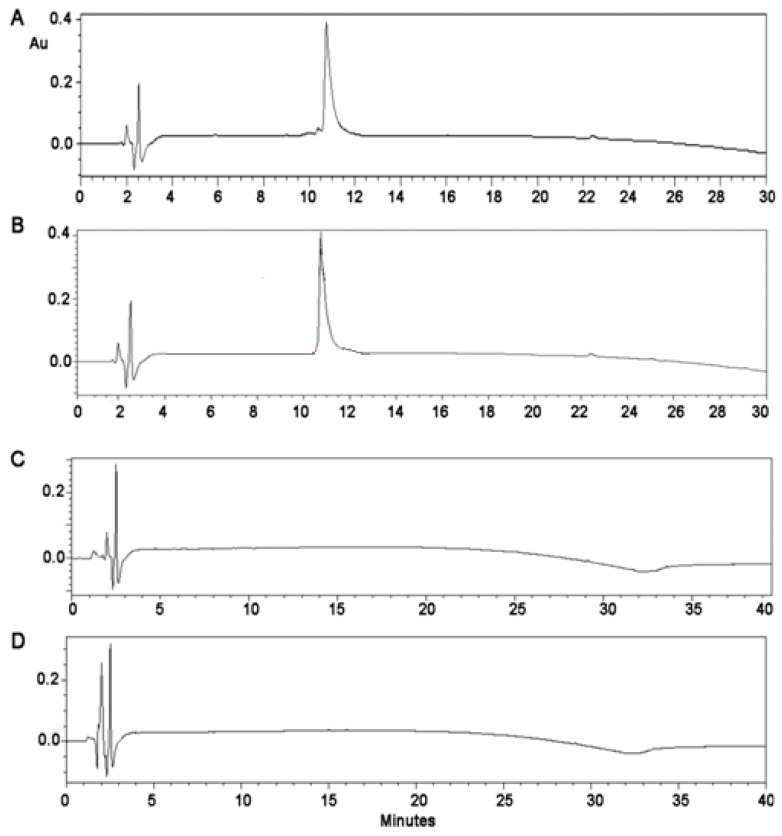
HPLC analysis of the attempted reaction between MOG_35–55_ and the oxidized mannan: (**A**) (KG)_5_-MOG_35–55_; (**B**) reaction mixture of the MOG_35–55_ and the oxidized mannan—no reaction was evident from the MOG_35–55_ peak at the retention time 10.7; (**C**) mannan; (**D**) oxidized mannan.

The validity of the total conjugation of the MOG_35–55_ peptide through a linker (KG)_5_ to oxidized mannan and the stability of the (KG)_5_-MOG_33–55_ peptide over the time was confirmed when conjugation of the MOG_35–55_ peptide with mannan was attempted with or without the use of the (KG)_5_ linker. As shown in the HPLC graphs (Figure 4), which depict the status of the MOG_35–55_ peptide while steering with oxidized mannan, no conjugation was recorded. The HPLC, which appeared at rt 10.75, indicates that the peptide without the (KG)_5_ linker was not-conjugated to oxidized mannan. This was attributed to the lack of the (KG)_5_ linker sequence and, consequently, to the lack of the Lys amino groups of the (KG)_5_ linker, that are required to react with the aldehyde groups of the oxidized mannan.

### 3.3. Enzymatic Cleavage of the Peptide (KG)_5_-MOG_35–55_ Results to the Fragment MOG_37–55_

**Figure 5:**
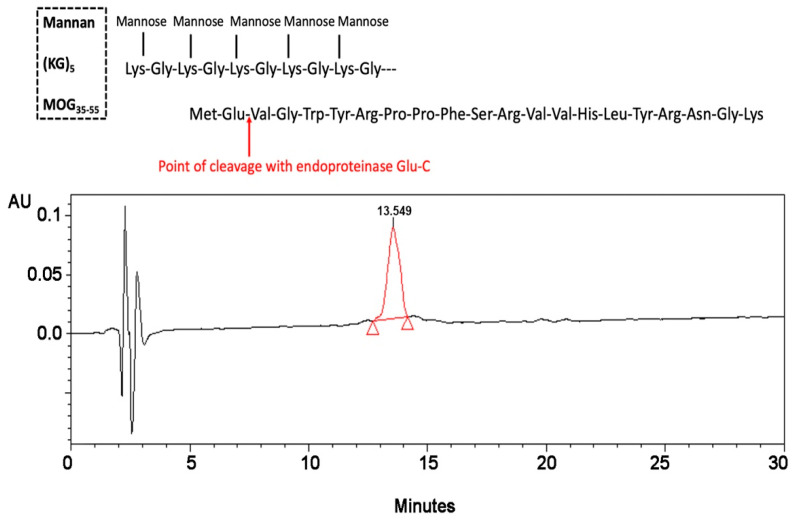
Control experiments using endo-proteinase Glu-C to cleave the peptide between Glu(36) and Val(37) of (KG)_5_-MOG_35–55_. An HPLC chromatogram for the MOG_37–55_ peptide analogue (concentration 0.5 mg/mL in 100 mM Tris-HCl, pH 7.8, after enzymatic treatment with endo Glu). The fragment MOG_37–55_ was confirmed by mass spectroscopy.

**Figure 6:**
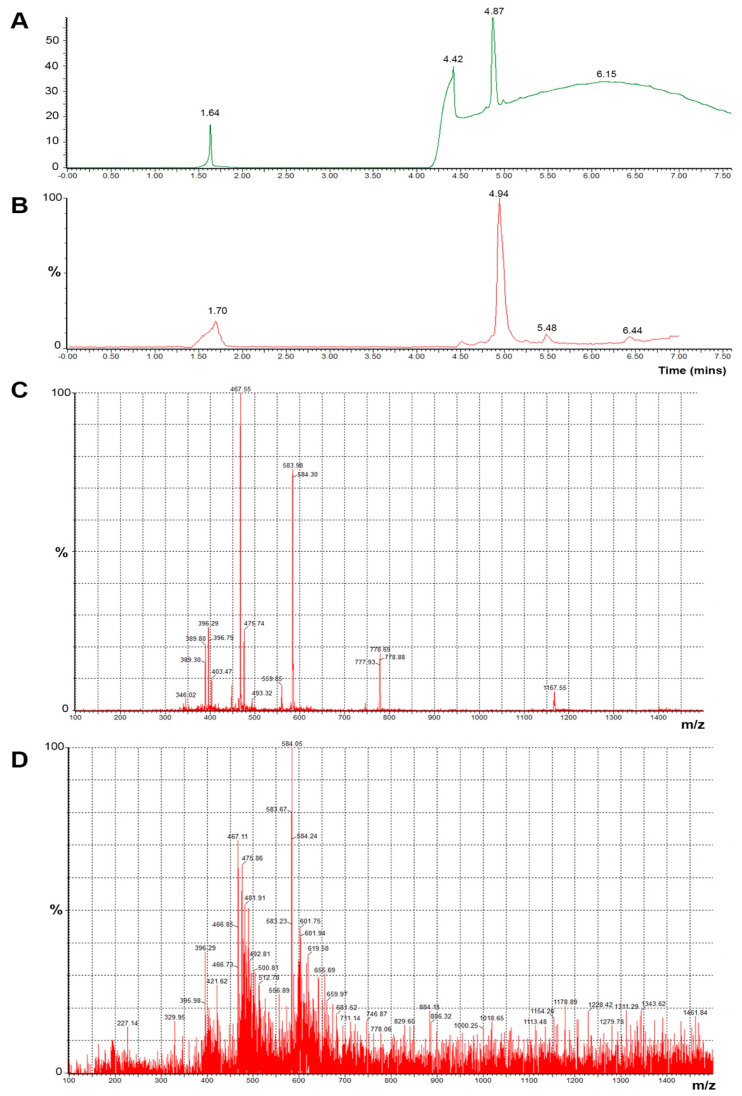
(**A**) (KG)_5_-MOG_35–55_ concentration 0.5 mg/mL after treatment with Endo-Glu-C in carbonate buffer in the UPLC analysis. The MOG_37–55_ fragment seen in the HPLC graph was confirmed by mass spectroscopy. (**B**) UPLC and MS analysis graphs of the sample (KG)_5_-MOG_35–55_ concentration 0.5 mg/mL after treatment with Endo-Glu-C in carbonate buffer. Mass spectrum of (**C**) the control MOG_37–55_ peptide and (**D**) the fragment of MOG_37–55_ isolated after enzymatic treatment with Glu-C endo-proteinase of the conjugated (KG)_5_-MOG_35–55_ with oxidized mannan. The fragment of MOG_37–55_ was confirmed by mass spectrum (mass ions: 396.29, 467.584, and 583.98).

To further confirm the validity of the findings showing complete conjugation of (KG)_5_-MOG_35–55_ with oxidized mannan, we carried out control experiments with enzymatic cleavage of the peptide between amino acids positions 36 and 37 occupied by amino acids Glu and Val, respectively. This cleavage experiment aimed to identify fragment MOG_37–55_ by HPLC analysis. The HPLC peak of the MOG_37–55_ peptide appeared at RT 13.55. Cleavage was achieved with endo glutamate peptidase enzyme (Sigma-Aldrich, St. Louis, MO, USA) by methods previously described [25,26,27]. In particular, endo-proteinase Glu-C V8, a proteolytic serine, sequencing grade from *Staphylococcus aureus* V8, (Roche, lot no. 32228800) was used. This enzyme acts at the carboxylic acid of the Glu amino acid. Enzyme cleavage was carried out using 100 mM Tris-HCL, pH 7.8, which has shown to allow the best cleavage performance. An HPLC peak that appeared at 13.55 min was confirmed to be a fragment of MOG_37–55_ by mass spectrometry. The same experiment of enzymatic hydrolysis with a glutamate enzyme was also carried out with oxidized mannan–(KG)_5_-MOG_35–55_ conjugate. A distinct peak at 13.55 min appeared due to the fragment MOG_37–55_ after the cleavage of peptide sequence between amino acids Glu(36) and Val(37) in the conjugate (Figure 5). The peak again was identified as fragment peptide of MOG_37–55_ by mass spectrometry after HPLC and UPLC-MS/MS. Figure 5 shows the cleavage of the peptide (KG)_5_-MOG_35–55_ by the endo-proteinase Glu-C and the fragment MOG_37–55_ confirmed by HPLC-MS and UPLC-MS/MS (Figure 5 and Figure 6).

### 3.4. Cyclic Voltammetry Showing Conjugation of (KG)_5_-MOG_35–55_ to Oxidized Mannan

**Figure 7:**
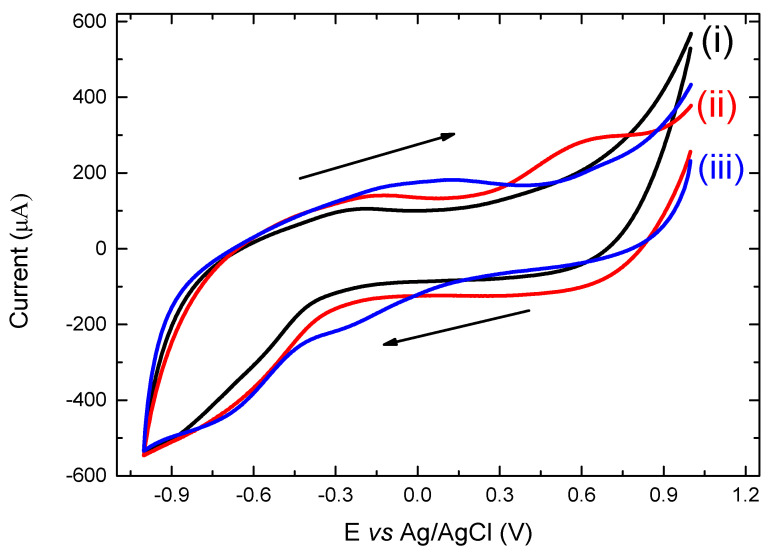
CV scans at a scan rate of 0.075 V s^−1^ of (i) a bare graphite/SiO_2_ film electrode; (ii) mannan and (iii) oxidized-mannan conjugated with (KG)_5_-MOG_35–55_.

The evaluation of the conjugate using cyclic voltammetry is presented in Figure 7. The CV of a bare graphite/SiO_2_ film electrode (i) does not show any reduction or oxidation peaks with the currents being limited by the conductivity of the graphite paste film. On the other hand, when mannan in 0.1 M buffer was added onto the surface of the film electrode (ii), an oxidation peak at approximately +0.57 V is noted. Furthermore, when 0.002 mg/mL of oxidized mannan–(KG)_5_-MOG_35–55_ conjugate was added (iii), the CV displayed a slight cathodic peak at −0.67 V, a clear cathodic peak at −0.18 V, and a characteristic broad anodic peak at +0.1 V. The two cathodic peaks observed were due to the presence of lysines in the linker molecule (KG)_5_, as it contained five lysines and five glycines in its structure and were necessary in order to conjugate the peptide to oxidized mannan. Therefore, the cathodic peaks were due to the presence of lysines. The superfluity of the free (KG)_5_-MOG_35–55_ peptide that was not conjugated with oxidized mannan and which resulted in the final complex of the oxidized mannan–(KG)_5_-MOG_35–55_ conjugate, probably caused the broad oxidation peak to occur on the CV. These results correlate well with the differential pulse voltammetry (DPV) signals we presented in a recent study of ours for the same purpose [24]. However, it should be noted that this is a proof-of-concept study, and we plan in the near future to further study the quantification of this and other conjugates using both voltametric techniques.

### 3.5. Stability of the Conjugate and Its Sensitivity

**Figure 8:**
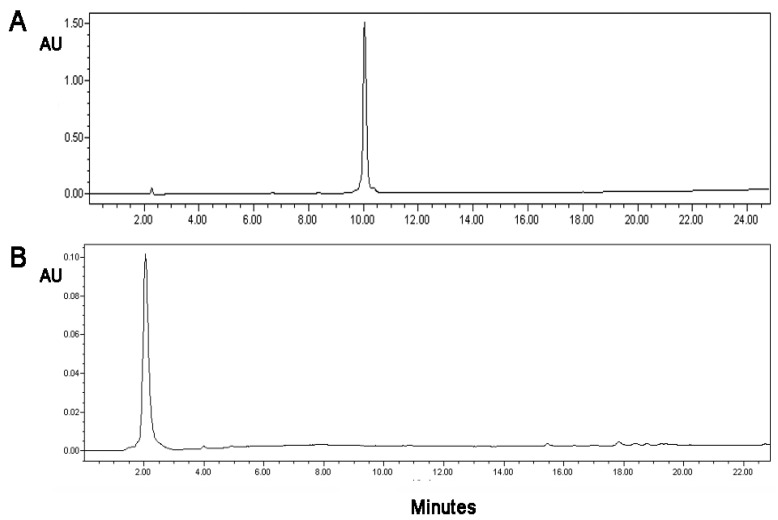
HPLC analysis of peptide (KG)_5_-MOG_35–55_ (214 nm) and oxidized mannan–(KG)_5_-MOG_35–55_. HPLC analysis of (**A**) (KG)_5_-MOG_35–55_ and (**B**) oxidized mannan–(KG)_5_-MOG_35–55_ solution after 3 years (214 nm) showing the stability of the conjugate. No free (KG)_5_-MOG_35_-_55_ peak or fragmentation was observed. The oxidized mannan–(KG)_5_-MOG_35–55_ conjugate was very polar, and it appeared at an earlier retention time compared to the less polar (KG)_5_-MOG_35–55_ peptide. The HPLC method used: gradient 5–100% AcN in 30 min.

**Figure 9:**
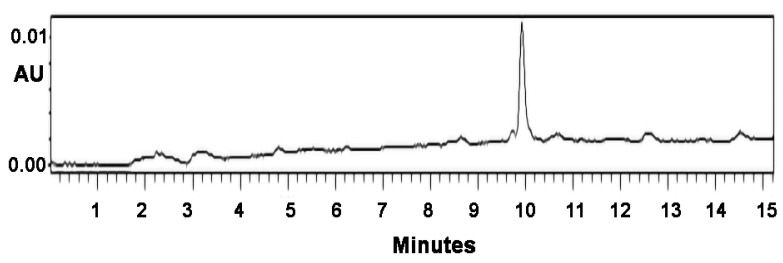
HPLC graph of the peptide (KG)_5_-MOG_35–55_ at 214 nm, showing a detection of the peptide at a low concentration of 2 μg/mL. The initial concentration of detection was 2 mg/mL.

**Figure 10:**
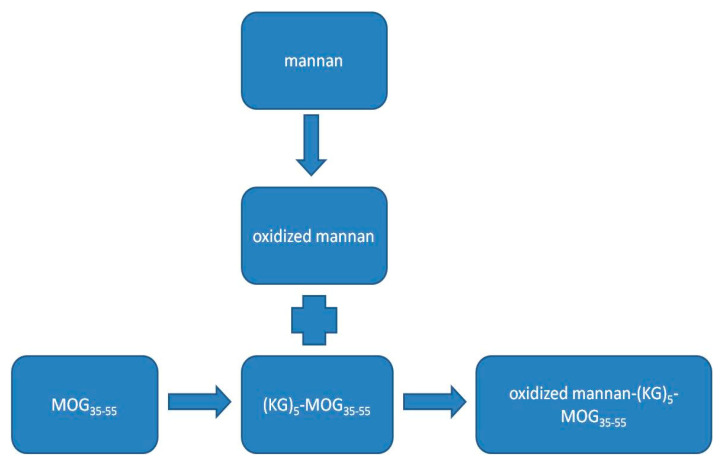
Oxidation of the mannan and the conjugation with the epitope peptide MOG_35–55_ linked with the linker (KG)_5_.

The stability of the oxidized mannan–(KG)_5_-MOG_35–55_ conjugate was analyzed after 3 years of storage. No peptide cleavage or detachment was noted by HPLC, as there were no signs of peptide fragmentation. In addition, old lab batches of oxidized mannan–(KG)_5_-MOG_35–55_ conjugate kept at 5 ± 3 °C since 2014 also did not show any signs of fragmentation or detachment during this period, indicating the stability of the conjugate (not shown). Hence, the conjugates were stable over a long time at both −20 °C and +5 ± 3 °C (Figure 8). This experiment provides insights into the stability of oxidized mannan–(KG)_5_-MOG_35–55_ over an extended period and HPLC method as a valid tool to monitor the conjugation, and confirms the stability of the conjugate over time. Figure 9 shows the sensitivity of the HPLC method, where 2 μg of (KG)_5_-MOG_35–55_ peptide was detected. The graphs show complete conjugation and no fragmentation or peptide cleavage. Figure 10 shows the overall scheme with the distinct steps from mannan and antigen peptide to the conjugate.

## 4. Discussion

### 4.1. Mannan as a Carrier

Mannan (a poly mannose), as a carrier for antigen delivery to dendritic cells, was first described by Apostolopoulos et al. in the early 1990s for cancer vaccine studies to stimulate appropriate immune responses [21,28]. Mannan is conjugated to proteins or peptides in its oxidized (comprising aldehydes) or reduced (aldehydes reduced to alcohols) form, and both bind to the mannose receptor efficiently; however, the stimulation of cytokines secreted by dendritic cells varies considerably, depending on the mode of conjugation. Mannan’s carrier/adjuvant properties make it an attractive material for biomedical applications such as the development of vaccines. Mannan covalently linked to a myelin peptide, as an example in this study, through the use of the linker (KG)_5_ on the N-terminus of MOG_35–55_ peptide, it selectively targets the mannose receptor on dendritic cells resulting in stimulation and immune regulation of the immune system.

### 4.2. The Role of the Linker (KG)_5_ for Conjugation

Our team was the first to introduce the decapeptide (KG)_5_ as a linker for the conjugation of antigenic peptides. Initially, large immunogenic proteins, such as keyhole limpet hemocyanin (KLH) and bovine serum albumin (BSA), were used as linkers between peptides and mannan due to their large number of lysines. KLH is a large protein containing 300–600 lysines, while BSA is a smaller protein containing 59 lysine residues. The advantage of using a small decapeptide (KG)_5_ containing only five lysines compared to the two large proteins containing a vast number of lysines is the elimination of unwanted immune activation resulting from the two proteins and their many epitopes. Herein, the MOG_35–55_ peptide’s conjugation to oxidized mannan was performed via the (KG)_5_ linker. As demonstrated, this approach provides a simple and efficient reaction via Schiff’s base formation between aldehyde groups of oxidized mannan and amino groups of lysine sidechains of the (KG)_5_-MOG_35–55_ peptide. In our studies using linker KGs of varying length (KG)*_n_*_=1–5_, we noted that the length of the linker plays a very critical role in the ability of the MOG_35–55_ peptide to be efficiently conjugated to the oxidized mannan scaffold. Naturally, there is only one lysine in the epitope peptide. It was found that five lysines, such as in the linker (KG)_5_, were the optimal number for complete mannan conjugation. As the length of the linker decreases from *n* = 5 to *n* = 1, conjugation also gradually decreased due to the steric hindrance and did not occur when *n* = 1 (data not shown). On the contrary, conjugation was complete when *n* = 5, revealing that the length of the linker and the number of Lys residues are important for maximum bio-conjugation.

### 4.3. Main Properties of Peptide Conjugate Excipients

The excipient of the conjugates was based mainly on polymannose mannan (already approved by the FDA in mannan-based cancer vaccines) [28]. In particular, the excipient: (i) is easy to manufacture, (ii) an inexpensive component, (iii) related to mannan-based vaccines for cancer that are already cleared by the FDA for human use, (iv) can possibly require only a few injections, which permanently modulate the immune system, and (v) is compatible with a large range of APIs. Furthermore, mannan from Saccharomyces Cerevisiae (i) carries the peptide connected with mannose through the (KG)_5_ linker to dendritic cells, (ii) contains unreactant aldehyde groups necessary for the immunoregulation of the dendritic cells, and (iii) contains intact mannose units, not oxidized, which are necessary to bind to the mannose receptors of the dendritic cells.

### 4.4. Mannan–Peptide Conjugate: Requirements for Immunoregulation of Dendritic Cells

In this oxidized mannan–(KG)_5_-MOG_35–55_ conjugate, the mannan platform delivers the peptide to antigen-presenting cells (i.e., dendritic cells and macrophages). The matrix contains non-reacted aldehyde groups necessary to allow the escape of peptides from endosomes within dendritic cells. The matrix also contains intact mannose residues, not oxidized, which are necessary to bind to the mannose receptors of dendritic cells as well as to Toll-like receptor 4 for stimulation of dendritic cells and immune responses (Figure 1).

### 4.5. Antigen Presentation by Tolerogenic Dendritic Cells Using the MOG_35–55_–Mannan Conjugate

A recent study showed that the pathogenic mechanism of MS is orchestrated mainly by autoreactive T and B cells that escape the mechanisms of central and peripheral immunological tolerance. The induction of immunological tolerance through the action of dendritic cells is a newly introduced strategy for the treatment of MS. Peripheral blood mononuclear cells (PBMCs) were isolated from patients with relapsing-remitting (RR-MS) and age- and sex-matched healthy individuals (controls). Inhibition of activation in the co-cultures with tolerogenic DCs, which presented the MOG_35–55_–mannan conjugate, was observed from the estimation of activation levels of CD4 T cells. Our results suggest that tolerogenic DCs loaded with the MOG_35–55_–mannan conjugate, which we generated in vitro, induce T-cell tolerance and can be used as a therapeutic vaccine for MS [29,30]. The novelty and applicability of the epitope mannan approach was confirmed in clinical cancer [21] and preclinical MS [20,30] studies.

### 4.6. Biological Activity of Mannan–Peptide Conjugates

Several biological studies, including in vitro and in vivo assays, have been carried out to evaluate the effects of mannan–myelin peptide conjugates [18,19]. Of interest, mannan–(KG)_5_-MOG_35–55_ conjugates showed protection in experimental autoimmune encephalomyelitis (EAE) therapeutic and prophylactic models and were associated with reduced antigen-specific T-cell proliferation but no alterations in Th1, Th17, and regulatory T-cell differentiation or T-cell apoptosis compared to EAE controls. In another study, mannan–MOG_35–55_ reversed experimental autoimmune encephalomyelitis, inducing a peripheral type 2 myeloid response, reduced CNS inflammation, and preserved axons in spinal cord lesions. More recently, a deaminated MOG_35–55_ peptide. Namely. modification of Asn(53) to Asp(53) as well as the non-deaminated peptide conjugated to oxidized mannan via (KG)_5_ were shown to inhibit the development of neurological symptoms and inflammatory demyelinating spinal cord lesions in EAE models.

### 4.7. Advantages of HPLC Methods Confirming Conjugation and the Stability of Peptide Conjugates

The advantages of HPLC methods that confirm conjugation and stability are summarized as follows: (i) The importance of the linker (KG)_5_ for conjugation is confirmed by a simple and facile HPLC analysis. Without (KG)_5_, mannan bio-conjugation does not proceed, and regulatory activity is lost. (ii) A novel enzymatic cleavage method by specific hydrolysis between amino acids Glu–Val was applied using enzyme endoglucanase to produce distinct fragments; in this case, the fragment MOG_37–55_ was seen in HPLC as a distinct peak. This fragment was identical with the control synthetic peptide MOG_37–55_, verifying that conjugation and stability are not possible by other methods for conjugates. (iii) A simple method confirming complete conjugation of the peptide to mannan, which is a requirement for approval of clinical trials of peptide conjugates, was applied. Complete conjugation allows for quantification of the peptide within the conjugate. (iv) An efficient method for evaluating the stability of the peptide conjugated mannan by monitoring the HPLC status of the solution was applied. Any peptide cleavage could be detected by HPLC even after years. Interestingly, samples kept in the fridge and at room temperature, even after a long time, confirmed the stability of the conjugate. (v) This method showed that the conjugation is complete within six hours, confirming that completion of conjugation is fairly quick.

### 4.8. Analysis by HPLC Techniques

The efficiency of HPLC is demonstrated by monitoring the covalent conjugation of peptides with mannan for subsequent biomedical applications. HPLC chromatography and amino acid analysis have been used mainly to provide a direct measurement of the amount of conjugated peptide to the polysaccharide mannan scaffold. The fast separation and absence of sample preparation provided by HPLC enable accurate yet real-time monitoring of the peptide conjugation process. HPLC can separate and quantify the different components of the reaction mixture as well as the (non-conjugated, free) peptide and the chemical coupling agents. In the example herein, a (KG)_5_-MOG_35–55_ peptide through (KG)_5_ was completely conjugated to the mannan as confirmed by all used HPLC and MS/MS techniques. In this study, the use of HPLC furthermore confirmed the complete conjugation and the quantification method as initially shown in the HPLC analysis. HPLC is a gold standard analytical tool that is user friendly, available in almost any laboratory, and has been accepted by all regulatory bodies. Hence, characterizing the completion of the conjugation’s reaction and assessing its stability and fragments using this single piece of equipment thus carries great value from a vaccine development and regulatory perspective.

### 4.9. Cyclic Voltammetry

Electrochemical voltametric techniques were also used to evaluate the conjugation efficiency of MS peptide-carrier conjugates. Graphite/SiO_2_ film electrodes and HPLC methods were previously shown by our group to be efficient in detecting drug molecules such as losartan. In this study, we used these methods to detect the conjugation efficiency of a peptide from the immunogenic region of myelin oligodendrocyte to a carrier, mannan. We plan in the near future to use voltametric techniques to quantify this and other conjugates and determine their limit of detection.

## 5. Conclusions

This work aimed to develop an analytical method for the quantification of mannan–peptide conjugates, which have shown to be good candidates for vaccines/immunotherapeutics against MS, and to investigate the role of (KG)_5_. Towards clinical evaluation in humans and obtaining approval for clinical trials of mannan–peptide conjugates, it is an important prerequisite to quantify the peptide within the conjugate or any potential degradants over time. As described here, a simple HPLC method was developed to quantify the peptide in the conjugated vaccine system over an extended period at −20 °C and 5 ± 3 °C. This method is in line with previous capillary electrophoresis experiments that also showed complete conjugation of myelin peptides with the mannan scaffold. The validity of the method was confirmed by the Glu-C endopeptidase enzyme which hydrolyzed the (KG)_5_-MOG_35–55_ peptide and the conjugate-oxidized mannan–(KG)_5_-MOG_35–55_ leading to the fragment MOG_37-55_ of MOG_35–55_, which is shown as an HPLC peak. Overall, the use of HPLC and UPLC-MS/MS was valid for the quantification of the peptide in the conjugate and the study of its stability over time. These methods can also be applicable to other peptide–carrier conjugations for their characterization. Overall, application of the myelin epitope-mannan approach allows the immunoregulation in MS patients through vaccination.

## Data Availability

The data presented in this study are available upon request from the corresponding author.

## References

[1] Steinman M.D.L. (1996). Multiple Sclerosis: A Coordinated Immunological Attack against Myelin in the Central Nervous System. Cell.

[2] Steinman L. (2001). Multiple sclerosis: A two-stage disease. Nat. Immunol..

[3] Kappos L., Comi G., Panitch H., Oger J., Antel J., Conlon P., Steinman L., Comi G., Kappos L., Oger J. (2000). Induction of a non-encephalitogenic type 2 T helper-cell autoimmune response in multiple sclerosis after administration of an altered peptide ligand in a placebo-controlled, randomized phase II trial. Nat. Med..

[4] Piatek P., Namiecinska M., Domowicz M., Przygodzka P., Wieczorek M., Michlewska S., Lewkowicz N., Tarkowski M., Lewkowicz P. (2019). MS CD49d+CD154+ Lymphocytes Reprogram Oligodendrocytes into Immune Reactive Cells Affecting CNS Regeneration. Cells.

[5] Apostolopoulos V., Matsoukas J. (2020). Advances in Multiple Sclerosis Research–Series I. Brain Sci..

[6] Gasperi C., Andlauer T.F.M., Keating A., Knier B., Klein A., Pernpeintner V., Lichtner P., Gold R., Zipp F., Then Bergh F. (2020). Genetic determinants of the humoral immune response in MS. Neurol.—Neuroimmunol. Neuroinflammation.

[7] Häusler D., Hajiyeva Z., Traub J.W., Zamvil S.S., Lalive P.H., Brück W., Weber M.S. (2020). Glatiramer acetate immune modulates B-cell antigen presentation in treatment of MS. Neurol.—Neuroimmunol. Neuroinflammation.

[8] Haines J.D., Inglese M., Casaccia P. (2011). Axonal Damage in Multiple Sclerosis. Mt. Sinai J. Med. A J. Transl. Pers. Med..

[9] Ziello A., Scavone C., Di Battista M.E., Salvatore S., Di Giulio Cesare D., Moreggia O., Allegorico L., Sagnelli A., Barbato S., Manzo V. (2021). Influenza Vaccine Hesitancy in Patients with Multiple Sclerosis: A Monocentric Observational Study. Brain Sci..

[10] Horwitz D.A., Fahmy T.M., Piccirillo C.A., La Cava A. (2019). Rebalancing Immune Homeostasis to Treat Autoimmune Diseases. Trends Immunol..

[11] Iberg C.A., Hawiger D. (2020). Natural and Induced Tolerogenic Dendritic Cells. J. Immunol..

[12] Zhou F., Ciric B., Zhang G.X., Rostami A. (2014). Immunotherapy using lipopolysaccharide-stimulated bone marrow-derived dendritic cells to treat experimental autoimmune encephalomyelitis. Clin. Exp. Immunol..

[13] Benedek G., Meza-Romero R., Jordan K., Keenlyside L., Offner H., Vandenbark A.A. (2015). HLA-DRα1-mMOG-35-55 treatment of experimental autoimmune encephalomyelitis reduces CNS inflammation, enhances M2 macrophage frequency, and promotes neuroprotection. J. Neuroinflammation.

[14] Tabansky I., Keskin D.B., Watts D., Petzold C., Funaro M., Sands W., Wright P., Yunis E.J., Najjar S., Diamond B. (2018). Targeting DEC-205−DCIR2+ dendritic cells promotes immunological tolerance in proteolipid protein-induced experimental autoimmune encephalomyelitis. Mol. Med..

[15] Boyko A., Therapontos C., Horakova D., Szilasiová J., Kalniņa J., Kolontareva J., Gross-Paju K., Selmaj K., Sereike I., Milo R. (2021). Approaches and challenges in the diagnosis and management of secondary progressive multiple sclerosis: A Central Eastern European perspective from healthcare professionals. Mult. Scler. Relat. Disord..

[16] Lee J.Y., Taghian K., Petratos S. (2014). Axonal degeneration in multiple sclerosis: Can we predict and prevent permanent disability?. Acta Neuropathol. Commun..

[17] Lublin F.D., Reingold S.C., Cohen J.A., Cutter G.R., Sorensen P.S., Thompson A.J., Wolinsky J.S., Balcer L.J., Banwell B., Barkhof F. (2014). Defining the clinical course of multiple sclerosis: The 2013 revisions. Neurology.

[18] Katsara M., Deraos G., Tselios T., Matsoukas M.-T., Friligou I., Matsoukas J., Apostolopoulos V. (2008). Design and Synthesis of a Cyclic Double Mutant Peptide (cyclo(87−99)[A91,A96]MBP87−99) Induces Altered Responses in Mice after Conjugation to Mannan: Implications in the Immunotherapy of Multiple Sclerosis. J. Med. Chem..

[19] Katsara M., Yuriev E., Ramsland P.A., Deraos G., Tselios T., Matsoukas J., Apostolopoulos V. (2008). Mannosylation of mutated MBP83–99 peptides diverts immune responses from Th1 to Th2. Mol. Immunol..

[20] Dagkonaki A., Avloniti M., Evangelidou M., Papazian I., Kanistras I., Tseveleki V., Lampros F., Tselios T., Jensen L.T., Möbius W. (2020). Mannan-MOG35-55 Reverses Experimental Autoimmune Encephalomyelitis, Inducing a Peripheral Type 2 Myeloid Response, Reducing CNS Inflammation, and Preserving Axons in Spinal Cord Lesions. Front. Immunol..

[21] Vassilaros S., Tsibanis A., Tsikkinis A., Pietersz G.A., McKenzie I.F.C., Apostolopoulos V. (2013). Up to 15-year clinical follow-up of a pilot Phase III immunotherapy study in stage II breast cancer patients using oxidized mannan–MUC1. Immunotherapy.

[22] Freedman M.S., Bar-Or A., Oger J., Traboulsee A., Patry D., Young C., Olsson T., Li D., Hartung H.P., Krantz M. (2011). A phase III study evaluating the efficacy and safety of MBP8298 in secondary progressive MS. Neurology.

[23] Warren K.G., Catz I., Ferenczi L.Z., Krantz M.J. (2006). Intravenous synthetic peptide MBP8298 delayed disease progression in an HLA Class II-defined cohort of patients with progressive multiple sclerosis: Results of a 24-month double-blind placebo-controlled clinical trial and 5 years of follow-up treatment. Eur. J. Neurol..

[24] Deskoulidis E., Petrouli S., Apostolopoulos V., Matsoukas J., Topoglidis E. (2020). The Use of Electrochemical Voltammetric Techniques and High-Pressure Liquid Chromatography to Evaluate Conjugation Efficiency of Multiple Sclerosis Peptide-Carrier Conjugates. Brain Sci..

[25] Drapeau G.R., Boily Y., Houmard J. (1972). Purification and properties of an extracellular protease of Staphylococcus aureus. J. Biol. Chem..

[26] Houmard J., Drapeau G.R. (1972). Staphylococcal Protease: A Proteolytic Enzyme Specific for Glutamoyl Bonds. Proc. Natl. Acad. Sci. USA.

[27] Klein T., Eckhard U., Dufour A., Solis N., Overall C.M. (2017). Proteolytic Cleavage—Mechanisms, Function, and “Omic” Approaches for a Near-Ubiquitous Posttranslational Modification. Chem. Rev..

[28] Apostolopoulos V., Pietersz G.A., Tsibanis A., Tsikkinis A., Drakaki H., Loveland B.E., Piddlesden S.J., Plebanski M., Pouniotis D.S., Alexis M.N. (2006). Pilot phase III immunotherapy study in early-stage breast cancer patients using oxidized mannan-MUC1 [ISRCTN71711835]. Breast Cancer Res..

[29] Rodi M., Dimisianos N., de Lastic A.-L., Sakellaraki P., Deraos G., Matsoukas J., Papathanasopoulos P., Mouzaki A. (2016). Regulatory Cell Populations in Relapsing-Remitting Multiple Sclerosis (RRMS) Patients: Effect of Disease Activity and Treatment Regimens. Int. J. Mol. Sci..

[30] Tseveleki V., Tselios T., Kanistras I., Koutsoni O., Karamita M., Vamvakas S.-S., Apostolopoulos V., Dotsika E., Matsoukas J., Lassmann H. (2015). Mannan-conjugated myelin peptides prime non-pathogenic Th1 and Th17 cells and ameliorate experimental autoimmune encephalomyelitis. Exp. Neurol..

